# Endogenous AMPK acts as a detrimental factor in fulminant hepatitis via potentiating JNK-dependent hepatocyte apoptosis

**DOI:** 10.1038/cddis.2017.62

**Published:** 2017-03-02

**Authors:** Kai Hu, Xianqiong Gong, Qing Ai, Ling Lin, Jie Dai, Lu Cai, Rong Jiang, Pu Ge, Li Zhang

**Affiliations:** 1 Department of Pathophysiology, Chongqing Medical University, Chongqing, China; 2 Hepatology Center, Xiamen Hospital of Traditional Chinese Medicine, Xiamen, Fujian Province, China; 3 Department of Physiology, Chongqing Medical University, Chongqing, China; 4 Hospital of Chongqing University of Arts and Sciences, Chongqing, China; 5 Department of Pathogenic Biology, Chongqing Medical University, Chongqing, China; 6 Laboratory of Stem cell and Tissue Engineering, Chongqing Medical University, Chongqing, China

## Abstract

The energy sensor AMP-activated protein kinase (AMPK) is crucial for energy homeostasis. Recent studies have revealed that AMPK is involved in various energy-intensive pathological processes such as inflammation and apoptosis. The physiological functions of hepatic AMPK have been well studied, but the pathological significance of AMPK in liver disorders remains largely unknown. In the present study, the phosphorylation status and the roles of AMPK were investigated in mice with lipopolysaccharide (LPS)/d-galactosamine (D-Gal)-induced fulminant hepatitis. The experimental data indicated that the phosphorylation of hepatic AMPK increased in mice with LPS/D-Gal-induced fulminant hepatitis. Pretreatment with the AMPK inhibitor compound C enhanced the early production of pro-inflammatory cytokines but suppressed the late activation of the caspase cascade, reduced the number of TUNEL-positive cells, decreased the elevation of aminotransferases, alleviated the histological abnormalities and improved the survival rate of LPS/D-Gal-insulted mice. Pretreatment with compound C suppressed LPS/D-Gal-induced phosphorylation of JNK. Inhibition of JNK alleviated LPS/D-Gal-induced liver injury, but the level of p53 remained unchanged in mice exposed to LPS/D-Gal. Post-insult treatment with the AMPK activator A-769662 further increased the phosphorylation levels of AMPK and JNK, enhanced hepatocyte apoptosis and deteriorated liver injury, all of these effects could be reversed by co-administration of the AMPK inhibitor or JNK inhibitor. Interestingly, post-insult treatment with the AMPK inhibitor also resulted in beneficial outcomes. These data suggested that AMPK might be a late detrimental factor in LPS/D-Gal-induced hepatitis via potentiating JNK-dependent hepatocyte apoptosis and AMPK might become a pharmacological target for the intervention of fulminant hepatitis.

AMP-activated protein kinase (AMPK) is an evolutionarily conserved energy sensor that has central roles in maintaining energy homeostasis.^1, 2^ AMPK is composed of the catalytic *α* subunit and the regulatory *β*/*γ* subunits.^3^ AMPK is activated by binding AMP or ADP to the *γ* subunit under falling energy status and the phosphorylation of the Thr^172^ residue in the *α*-subunit is required for AMPK activation.^4^ Activated AMPK shuts down the energy-consuming metabolic pathway and switches on the energy-producing catabolic metabolism, thus preserving the cellular ATP level.^5^ In addition to its roles in metabolic regulation, AMPK also participates in the regulation of energy-intensive pathological responses such as inflammation, apoptosis and proliferation.^6, 7^ Therefore, AMPK has emerged as a promising pharmacological target driving extensive investigation.^6^

Since the liver is one of the metabolic centers with complex functions in nutrition and energy metabolism, the physiological roles of AMPK in the liver have been intensively studied.^8^ These studies have found that AMPK promotes glucose oxidation but suppresses gluconeogenesis in the liver via direct phosphorylation and transcriptional modulation of key metabolic enzymes.^9, 10^ In addition, AMPK also controls the metabolism of fats and proteins in the liver.^10^ Although the important physiological functions of AMPK have been well studied, the pathological significance of AMPK in liver disorders remains largely unknown.

The liver is usually in contact with various harmful factors and hepatocyte damage induced by pathogens, drugs and poisons is very common worldwide.^11^ In the most severe situation, fulminant hepatitis is still a life-threatening problem with a high incidence of mortality.^12^ In the present study, the pathological roles of AMPK in a mouse model with lipopolysaccharide (LPS)/d-galactosamine (D-Gal)-induced fulminant hepatitis were investigated. LPS/D-Gal-induced liver injury in mice is a well-established experimental hepatitis model.^13^ The hepatocyte damage in this model largely depends on the early production of detrimental inflammatory mediators such as tumor necrosis factor alpha (TNF-*α*), and these deleterious factors might induce massive hepatocyte apoptosis and lethal outcomes at the late stage.^14, 15^ In the present study, the phosphorylation status of AMPK was determined. Then the activation of AMPK was inhibited by a pharmacological inhibitor, and the degree of inflammation and apoptosis and the potential underlying mechanism were evaluated.

## Results

### Inhibition of AMPK alleviated liver injury

**Figure 1:**
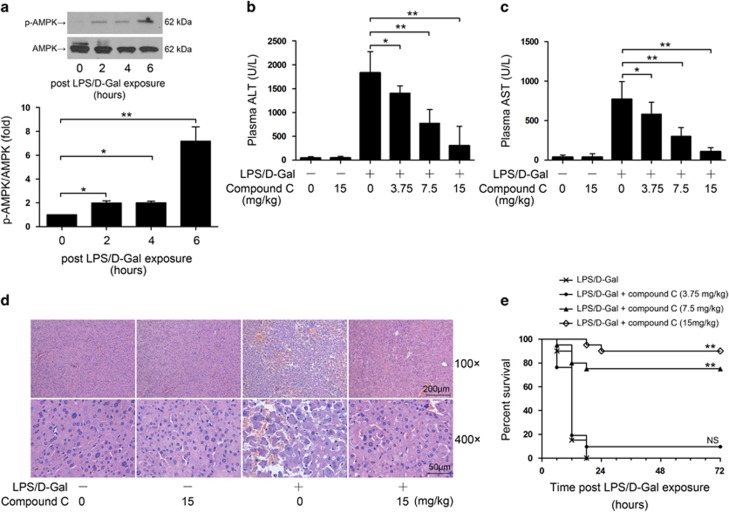
Inhibition of AMPK alleviated LPS/D-Gal-induced liver injury. (**a**) Mice were challenged with LPS/D-Gal to induce fulminant hepatitis, the level of phosphorylated AMPK*α* (Thr^172^) and the total level of AMPK*α* were determined by western blot analysis at various time points (0, 2, 4 and 6 h) after LPS/D-Gal exposure. Data were expressed as mean±S.D., *n*=4. (**b**–**d**) Mice were pretreated with various doses of the AMPK inhibitor compound C and then exposed to LPS/D-Gal, the liver and plasma samples were harvested at 6 h after LPS/D-Gal exposure. The levels of (**b**) ALT and (**c**) AST in plasma were determined, *n*=8. (**d**) Liver sections were stained with hematoxylin and eosin for morphological evaluation and the representative liver sections of each group are shown. (**e**) Another set of animals were exposed to LPS/D-Gal with or without compound C, the mortality of the animals was monitored and the percent survival rate was expressed as Kaplan–Meier survival curves, *n*=20. (**P<0.01)

In mice challenged with LPS/D-Gal, the phosphorylation level of AMPK gradually increased and the phosphorylation level of AMPK was higher at the late stage (Figure 1a). We next questioned whether the activation of AMPK was involved in the development of LPS/D-Gal-induced hepatitis. In the present study, compound C, a widely used AMPK inhibitor,^16, 17^ was administered before LPS/D-Gal exposure. The results indicated that pretreatment with compound C dose-dependently suppressed the elevation of plasma aminotransferases (ALT and AST) (Figures 1b and c). Consistently, the histopathological abnormalities induced by LPS/D-Gal were alleviated by compound C pretreatment (Figure 1d). To evaluate the overall outcomes, the accumulative survival rate of the experimental animals was monitored and the results indicated that pretreatment with compound C markedly improved the survival rate of LPS/D-Gal-insulted mice in a dose-dependent manner (Figure 1e).

These data suggested that inhibition of AMPK might result in beneficial outcomes in LPS/D-Gal-induced liver injury. Interestingly, our previous study found that pretreatment with the widely used AMPK activator, 5-Aminoimidazole-4-carboxamide-1-*β*-d-ribofuranoside (AICAR), alleviated the liver injury in LPS/D-Gal-exposed mice.^18^ The present study also observed the protective benefits of another AMPK activator, A-769662,^19, 20^ in this animal model (Supplementary Figure 1). Thus, both the AMPK inhibitor compound C and the two AMPK activators attenuated LPS/D-Gal-induced liver injury, but we speculated that their beneficial effects might be attributed to different mechanisms.

### Inhibition of AMPK enhanced the early inflammatory response

**Figure 2:**
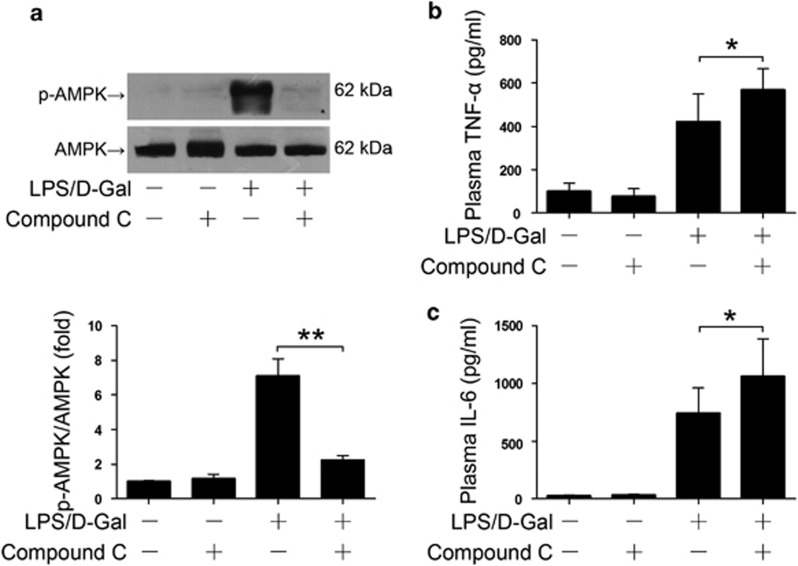
Activation of AMPK suppressed LPS/D-Gal-induced early inflammation. (**a**) Mice were exposed to LPS/D-Gal with or without AMPK inhibitor compound C pretreatment, the liver samples were harvested 6 h after LPS/D-Gal exposure, the level of phosphorylated AMPK*α* and the total level of AMPK*α* were determined by western blot analysis, *n*=4. (**b** and **c**) Another set of animals were killed 1.5 h after LPS/D-Gal exposure, the plasma samples were harvested and the level of (**b**) TNF-*α* and (**c**) IL-6 in plasma were determined by ELISA, *n*=8. (*P<0.05, **P<0.01)

The early production of pro-inflammatory cytokines, such as TNF-*α*, has crucial roles in the LPS/D-Gal model.^13^ The inflammatory response is an energy-intensive molecular event, which can be profoundly modified by AMPK.^21^ We next investigated whether the early production of TNF-*α* might be regulated by compound C. The results indicated that pretreatment with compound C decreased the phosphorylation level of AMPK (Figure 2a). In addition, compound C pretreatment resulted in increased expression of TNF-*α* (Figure 2b), a major detrimental pro-inflammatory factor in LPS/D-Gal-induced liver injury.^22, 23^ Consistently, the level of IL-6 in LPS/D-Gal-insulted mice was also elevated in mice that received compound C pretreatment (Figure 2c).

In contrast, pretreatment with the AMPK activator A-769662 resulted in enhanced phosphorylation of AMPK but decreased production of TNF-*α* (Supplementary Figure 2). Therefore, activation of AMPK might suppress the inflammatory response at the early stage after LPS/D-Gal challenge, which might contribute to the beneficial effects of the AMPK activators. However, the increased production of pro-inflammatory cytokines was inconsistent with the alleviated liver injury in the compound C-pretreated group. Therefore, another mechanism might underlie the protective effects of the AMPK inhibitor.

### Inhibition of AMPK suppressed hepatocyte apoptosis

**Figure 3:**
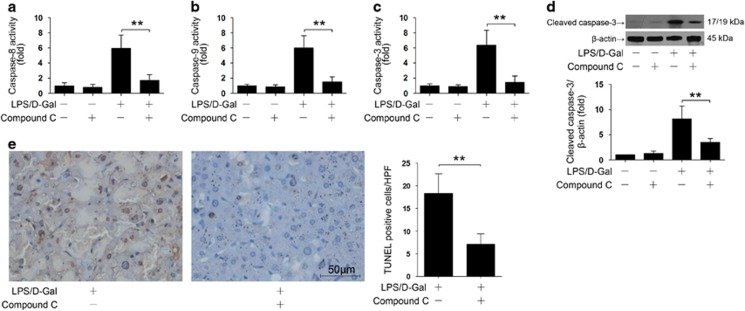
Inhibition of AMPK suppressed LPS/D-Gal-induced caspase activation and hepatocyte apoptosis. Mice were exposed to LPS/D-Gal with or without AMPK inhibitor compound C pretreatment, the liver samples were harvested 6 h after LPS/D-Gal exposure. The activities of (**a**) caspase-8, (**b**) caspase-9 and (**c**) caspase-3 were determined, *n*=8. (**d**) The level of cleaved caspase-3 were determined by western blot analysis, *n*=4. (**e**) The apoptotic cells were determined by TUNEL assay. Representative liver sections of each group are shown (original magnification 400). The numbers of TUNEL-positive cells in 10 randomly selected high-power field were counted under microscope, *n*=4. (**P<0.01)

The early production of the detrimental pro-inflammatory factors is followed by massive hepatocyte apoptosis in LPS/D-Gal-challenged mice.^13^ Because the protective benefits of AMPK inhibition could not be attributed to the enhanced early inflammatory response, we questioned whether AMPK inhibition could modulate hepatocyte apoptosis at the late stage. The experimental data indicated that pretreatment with compound C significantly inhibited the activation of caspase-8, −9 and −3 (Figures 3a–c). Western blot analysis also showed that the cleavage of caspase-3 was inhibited by compound C (Figure 3d). Consistently, the number of TUNEL-positive cells in LPS/D-Gal-insulted mice also decreased after compound C pretreatment (Figure 3e). These data suggested that AMPK might be involved in the progression of LPS/D-Gal-induced liver injury via promoting hepatocyte apoptosis at the late stage, while the protective benefits of the AMPK inhibitor compound C might result from suppressed hepatocyte apoptosis.

### JNK but not p53 was associated with the pro-apoptotic effects of AMPK

**Figure 4:**
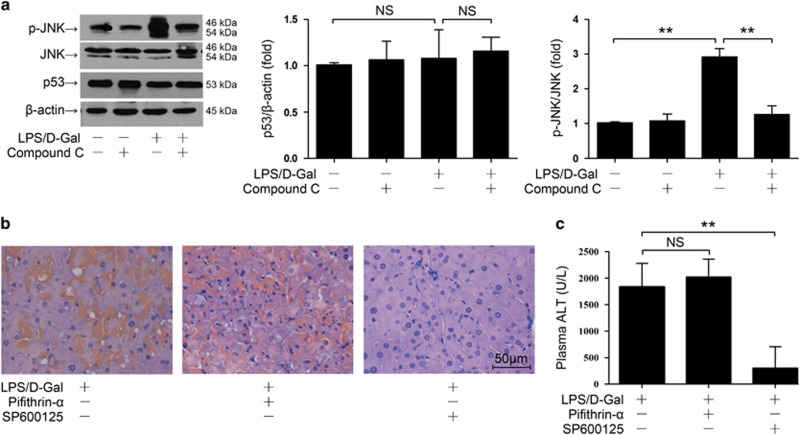
JNK but not p53 was associated with the pro-apoptotic properties of AMPK. (**a**) Mice were exposed to LPS/D-Gal with or without AMPK inhibitor compound C pretreatment, the liver samples were harvested 6 h after LPS/D-Gal exposure. The level of phosphorylated JNK, total JNK and p53 were determined by western blot analysis, *n*=4. (**b** and **c**) Mice were exposed to LPS/D-Gal with or without the p53 inhibitor pifithrin-*α* or JNK inhibitor SP600125, the liver and plasma samples were harvested 6 h after LPS/D-Gal exposure. (**b**) Liver sections were stained with hematoxylin and eosin for morphological evaluation and the representative liver sections of each group are shown. (**c**) The levels of ALT in plasma were determined, *n*=8. (^NS^*P*>0.05, *P<0.05, **P<0.01)

P53 and JNK have been reported to be the representative apoptosis-related targets of AMPK.^24, 25^ In the present study, the accumulation of p53 and the phosphorylation of JNK were determined. Western blot analysis found that the level of p53 remained unchanged after LPS/D-Gal exposure but LPS/D-Gal exposure induced significant phosphorylation of JNK (Figure 4a). Inhibition of AMPK had no obvious effects on p53 level but significantly suppressed LPS/D-Gal-induced JNK phosphorylation (Figure 4a). The experimental data also indicated that inhibition of p53 had no obvious effects on LPS/D-Gal-induced morphological abnormality or ALT elevation (Figures 4b and c). However, inhibition of JNK by SP600125 significantly alleviated the histological abnormalities and decreased the ALT level (Figures 4b and c). These data suggested that JNK but not p53 might be associated with the pro-apoptotic effects of AMPK in LPS/D-Gal-induced hepatitis.

### The pro-apoptotic effects of AMPK depended on JNK

**Figure 5:**
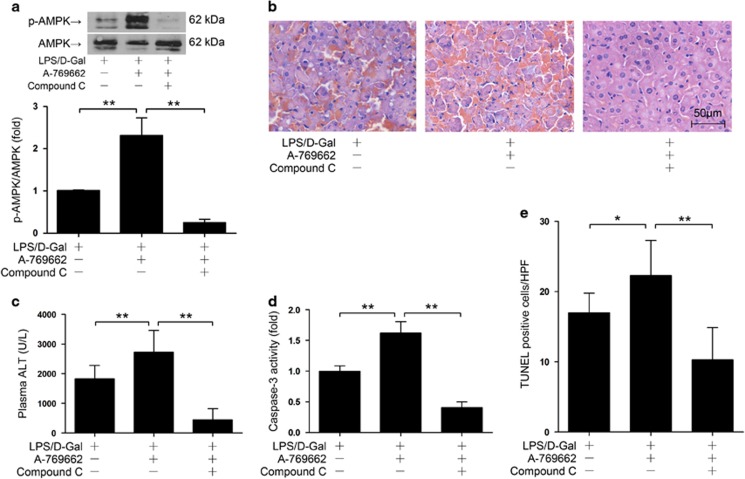
Post-insult activation of AMPK promoted hepatocyte apoptosis and deteriorated liver injury. Mice were exposed to LPS/D-Gal, AMPK activator A769662 or combined with AMPK inhibitor compound C was administrated at 2 h after LPS/D-Gal exposure, the liver and plasma samples were harvested at 6 h after LPS/D-Gal exposure. (**a**) The level of phosphorylated AMPK and total AMPK were determined by western blot analysis, *n*=4. (**b**) Liver sections were stained with hematoxylin and eosin for morphological evaluation and the representative liver sections of each group are shown. (**c**) The level of ALT in plasma and (**d**) the activity of caspase-3 in liver was determined, *n*=8. (**e**) The apoptotic cells were determined by TUNEL assay and the numbers of TUNEL-positive cells in 10 randomly selected high-power field were counted under microscopy, *n*= 4. (*P<0.05, **P<0.01)

To further confirm the pro-apoptotic effects of AMPK, the AMPK activator was administered post insult. We found that post-treatment with the AMPK activator A-769662 2 h after LPS/D-Gal exposure further increased the phosphorylation level of AMPK in LPS/D-Gal-insulted mice (Figure 5a). As expected, post-insult AMPK activation enhanced the activity of caspase-3, increased the number of TUNEL-positive cells, raised the level of ALT and deteriorated the histological abnormalities (Figures 5b–e). All of the above alterations were reversed after AMPK inhibitor administration (Figures 5a–e). These data provided evidence to support the hypothesis that activation of AMPK at the late stage might promote LPS/D-Gal-induced hepatocyte apoptosis and liver injury.

**Figure 6:**
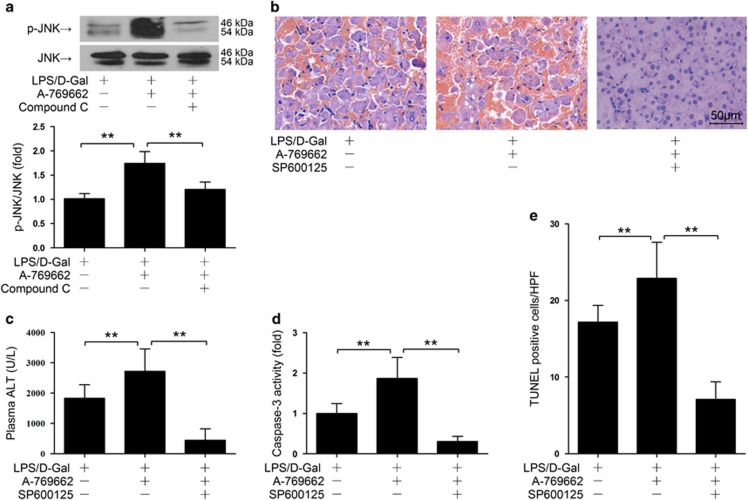
Post-insult activation of AMPK promoted liver injury via JNK. (**a**) Mice were exposed to LPS/D-Gal, AMPK activator A769662 or combined with AMPK inhibitor compound C was administrated at 2 h after LPS/D-Gal exposure, the liver samples were harvested 6 h after LPS/D-Gal exposure. The level of phosphorylated JNK and total JNK were determined by western blot analysis, *n*=4. (**b**–**e**) Mice were exposed to LPS/D-Gal, AMPK activator A769662 or combined with JNK inhibitor SP600125 was administrated at 2 h after LPS/D-Gal exposure, the liver and plasma samples were harvested at 6 h after LPS/D-Gal exposure. (**b**) Liver sections were stained with hematoxylin and eosin for morphological evaluation and the representative liver sections of each group are shown. (**c**) The level of ALT in plasma and (**d**) the activity of caspase-3 in liver was determined, *n*=8. (**e**) The apoptotic cells were determined by TUNEL assay and the numbers of TUNEL-positive cells in 10 randomly selected high-power field were counted under microscope, *n*= 4. (*P<0.05, **P<0.01)

We then questioned whether the pro-apoptotic effects of AMPK were mediated by JNK. The present study found that post-insult activation of AMPK by A769662 enhanced LPS/D-Gal-induced phosphorylation of JNK (Figure 6a), which was reversed by the AMPK inhibitor compound C (Figure 6a). These data suggested that JNK phosphorylation was modulated by AMPK. The present study also found that the enhanced JNK phosphorylation, increased hepatocyte apoptosis and deteriorated liver injury in AMPK activator-treated mice were reversed by JNK inhibition (Figures 6a and e). These data suggested that the pro-apoptotic effects of AMPK might depend on JNK.

### Post-treatment with the AMPK inhibitor provided therapeutic benefits

**Figure 7:**
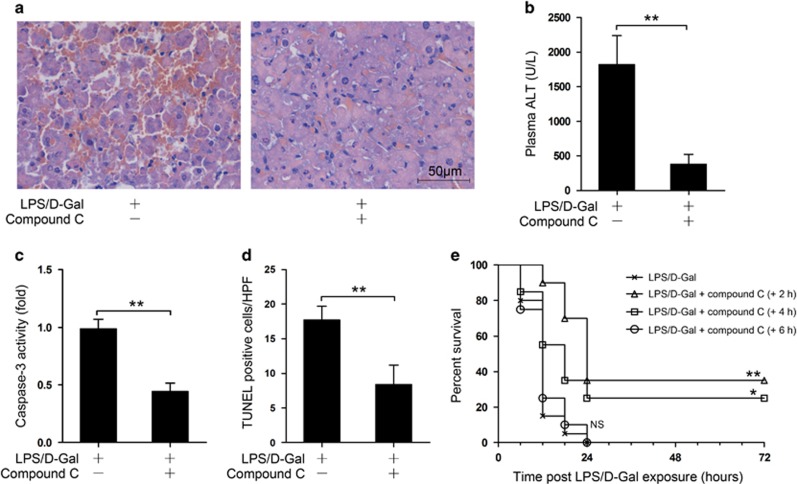
Post-insult treatment with the AMPK inhibitor provided therapeutic benefits. (**a**–**d**) Mice were exposed to LPS/D-Gal, and the AMPK inhibitor compound C was administrated 2 h after LPS/D-Gal exposure, the liver and plasma samples were harvested at 6 h after LPS/D-Gal exposure. (**a**) Liver sections were stained with hematoxylin and eosin for morphological evaluation and the representative liver sections of each group are shown. (**b**) The level of ALT in plasma and (**c**) the activity of caspase-3 in the liver were determined, *n*=8. (**d**) The apoptotic cells were determined by TUNEL assay and the numbers of TUNEL-positive cells in 10 randomly selected high-power field were counted under microscope, *n*=4. (**e**) Another set of animal were exposed to LPS/D-Gal with administration of compound C at various time points after LPS/D-Gal exposure, the mortality of the animals was monitored and the percent survival rate was expressed as Kaplane–Meier survival curves, *n*=20. (**P<0.01)

Finally, we questioned whether post-insult treatment with compound C could also protect against liver injury. Interestingly, we found that post-treatment with compound C significantly alleviated the histopathological abnormalities, decreased the level of ALT, suppressed the activity of caspase-3 and reduced the number of TUNEL-positive cells in LPS/D-Gal-insulted mice (Figures 7a and e). In addition, post-treatment with compound C also reduced the mortality rate of LPS/D-Gal-insulted mice (Figure 7e). These data suggested that post-treatment with the AMPK inhibitor could also provide therapeutic benefits.

## Discussion

AMPK is an AMP/ADP sensitive kinase with multiple physiological functions.^4^ Recent studies have found that AMPK was involved in the development of cancer, cardiac fibrosis, Huntington's disease and so on.^26, 27, 28^ In the present study, we found that AMPK was activated in mice with LPS/D-Gal-induced fulminant hepatitis. Pharmacological inhibition of AMPK by compound C significantly suppressed the elevation of plasma aminotransferases, alleviated the histological alterations and increased the survival rate of experimental animals. These data suggested that activation of AMPK might be a detrimental molecular event involved in the progression of LPS/D-Gal-induced fulminant hepatitis.

The liver injury induced by LPS/D-Gal in mice largely depends on the production of the pro-inflammatory and pro-apoptotic cytokine TNF-*α*, which usually reaches its peak level 1.5 h after LPS exposure.^29^ Then, the high level of TNF-*α* induces massive apoptosis of hepatocytes via the death receptor-dependent pathway.^30^ Therefore, both inhibition of TNF-*α* production at the early stage and suppression of TNF-*α*-mediated pro-apoptotic signaling at the late stage might be rational strategies for the prevention of LPS/D-Gal-induced hepatitis. Actually, a great number of experimental studies have observed the beneficial effects of anti-inflammatory reagents or anti-apoptotic compounds in the LPS/D-Gal model.^31, 32, 33, 34^

In the present study, inhibition of AMPK by compound C resulted in increased expression of pro-inflammatory cytokines 1.5 h after LPS/D-Gal exposure but treatment with the AMPK activator A-769660 decreased the level of pro-inflammatory cytokines. Consistently, our previous study also observed the suppressive effects of the AMPK activator AICAR on the level of TNF-*α* at the early stage after LPS/D-Gal exposure.^18^ In agreement with these findings, the anti-inflammatory activities of AMPK have recently been confirmed *in vitro* and *in vivo*.^35^ Thus, AMPK might be a negative regulator that controls the inflammatory response at the early stage after LPS/D-Gal exposure. However, the increased production of pro-inflammatory cytokines was inconsistent with the alleviated liver injury in the AMPK inhibitor-pretreated group.

The present study also found that inhibition of AMPK by compound C significantly suppressed the activation of the caspase cascade and the appearance of TUNEL-positive cells. The pro-apoptotic activities of AMPK have been confirmed in keratinocytes, neurons, adipocytes and so on.^36, 37, 38^ Thus, AMPK might also have pivotal roles in regulating hepatocyte apoptosis at the late stage after LPS/D-Gal challenge. There are several factors associated with the modulatory activity of AMPK on apoptosis.^39^ The present study found that the pro-apoptotic property of AMPK was mediated by JNK but not p53. Consistent with our findings, several studies have found that JNK was the downstream target responsible for the pro-apoptotic action of AMPK.^24, 40^ Thus, AMPK might promote hepatocyte apoptosis at the late stage via activating JNK.

In the present study and our previous study, pretreatment with the AMPK activator or AMPK inhibitor both alleviated liver injury, but the underlying mechanisms might be completely different. Pretreatment with the AMPK activator promoted the activation of AMPK and suppressed the production of TNF-*α* at the early stage, which might subsequently result in impaired activation of the pro-apoptotic pathway at the late stage. In contrast, pretreatment with the AMPK inhibitor suppressed the activation of AMPK and increased the production of TNF-*α* at the early stage, but it strongly suppressed the pro-apoptotic signal at the late stage, which might also result in suppressed hepatocyte apoptosis and alleviated liver injury. In clinical settings, pre-insult administration of therapeutic drugs seems impractical under most situations, but it is exciting that post treatment with the AMPK inhibitor compound C could also result in suppressed hepatocyte apoptosis, alleviated histological abnormalities and improved survival rate. Therefore, the AMPK inhibitors, but not the AMPK activators, might have potential value for the clinical intervention of fulminant hepatitis.

Taken together, the present study found that AMPK was activated in mice with LPS/D-Gal-induced fulminant hepatitis and the activated AMPK promoted hepatocyte apoptosis via phosphorylating JNK at the late stage after LPS/D-Gal challenge, while inhibition of AMPK resulted in suppressed apoptosis and alleviated liver injury. Most importantly, post-treatment with the AMPK inhibitor could also provide therapeutic benefits. Thus, AMPK might have potential value for pharmacological intervention of fulminant hepatitis.

## Materials and methods

### Materials

LPS (from *Escherichia coli*, 055:B5), D-Gal and pifithrin-*α* were the products of Sigma (St. Louis, MO, USA). Compound C and A-769662 were the products of Cayman Chemical (Ann Arbor, MI, USA). SP600125 was the product of Enzo life sciences (New York, NY, USA). The kits for the determination of alanine aminotransferase (ALT) and aspartate aminotransferase (AST) were produced by Nanjing Jiancheng Bioengineering Institute (Nanjing, China). The enzyme-linked immunosorbent assay (ELISA) kits for determination of mouse TNF-*α* and interleukin 6 (IL-6) were produced by NeoBioscience Technology Company (Shenzhen, China). The total protein extract kit and the colorimetric assay kits for caspase-3, -8, -9 were produced by Beyotime Institute of Biotechnology (Jiangsu, China). The *In Situ* Cell Death Detection Kit was produced by Roche (Indianapolis, IN, USA). The rabbit anti-mouse AMPK, phospho-AMPK*α* (Thr^172^), c-Jun N-terminal kinase (JNK), phospho-JNK (Thr^183^/ Thr^185^), cleaved caspase-3 and *β*-actin antibodies were the products of Cell Signaling Technology (Danvers, MA, USA). The BCA protein assay kit, the horseradish peroxidase-conjugated goat anti-rabbit antibody and the enhanced chemiluminescence (ECL) reagents were the products of Pierce Biotechnology (Rockford, IL, USA).

### Animals

Male BALB/c mice weighing 20–25 g were obtained from the Experimental Animal Center of Chongqing Medical University. The animals were housed in a specific pathogen-free room at a temperature of 20–25 °C and 50±5% relative humidity under a 12  h dark/light cycle. All animals were fed with a standard laboratory diet and water *ad libitum*. The mice were acclimatized for at least l week before use. The experimental procedures involving animals were approved by the Animal Care and Use Committee of Chongqing Medical University.

### LPS/D-Gal-induced liver injury

Hepatitis was induced in mice with intraperitoneal injection of LPS (10 *μ*g/kg) combined with D-Gal (700 mg/kg). The mice were killed at various time points (0, 2, 4 and 6 h) after LPS/D-Gal challenge, the liver samples were harvested and the phosphorylation level of AMPK was determined. To evaluate the potential roles of AMPK in hepatitis, the mice were treated pre-insult with various doses of the AMPK inhibitor compound C (3.75–15 mg/kg, dissolved in DMSO, i.p.) or treated post insult with the AMPK activator A-769662 (30 mg/kg, dissolved in NS, i.p.). To investigate the underlying mechanism, the p53 inhibitor pifithrin-*α* (2 mg/kg, dissolved in DMSO) or the JNK inhibitor SP600125 (50 mg/kg, dissolved in DMSO) was intraperitoneally injected before A-769662 administration. To determine the mortality, survival of the mice (*n*=20 per group) was assessed every 6 h for at least 7 days and the cumulative survival curve was depicted using the Kaplan–Meier method.

### Determination of liver enzymes

The levels of ALT and AST in plasma were determined using the colorimetric kits according to the manufacturer's instructions (Nanjing Jiancheng). The activity of ALT or AST was calculated based on the standard curve.

### Histological analysis

Formalin-fixed specimens were embedded in paraffin and stained with hematoxylin and eosin for histopathological analysis under a light microscope (Olympus, Tokyo, Japan).

### Determination of pro-inflammatory cytokines by ELISA

The levels of TNF-*α* and IL-6 in plasma were determined using the ELISA kits according to the manufacturer's instructions (NeoBioscience). The concentration of TNF-*α* or IL-6 was calculated based on the standard curve.

### Determination of caspase activities

The activities of caspase-3, -8 and -9 in liver samples were determined using the colorimetric assay kits according to the manufacturer's instructions (Beyotime). Briefly, the homogenates of the liver samples were prepared and centrifuged for 1 min at 10 000 × *g*. The supernatant was incubated with Ac-DEVD-pNA, Ac-IETD-pNA and Ac-LEHD-pNA substrates for determination of caspase-3, -8 and -9, respectively, for 90 min at 37 °C. The activities of caspases were calculated according to the absorbance measured at 405 nm and the value was normalized by the protein concentration of the same sample.

### Terminal deoxynucleotidyl transferase-mediated nucleotide nick-end labeling (TUNEL) assay

The apoptotic cells were visualized using the *In Situ* Cell Death Detection Kit according to the manufacturer's instructions (Roche). The terminal transferase reactions produced a dark-brown precipitate in the nuclei of apoptotic cells.

### Western blot analysis

The total proteins were prepared and the protein concentration was determined using the BCA protein assay kit. The protein extracts were then fractionated on polyacrylamide-SDS gel and transferred to nitrocellulose membrane. The membrane was blocked with 5% (w/v) nonfat milk in Tris-buffered saline containing 0.05% tween-20, and then the membrane was incubated with primary antibody against AMPK, phospho-AMPK*α*, JNK, phospho-JNK, cleaved capase-3 or *β*-actin overnight at 4 °C, followed by incubation with secondary antibody. Antibody binding was visualized with an ECL chemiluminescence system and short exposure of the membrane to X-ray films.

### Statistical analysis

The experimental data were expressed as mean±S.D. and the statistical significance was determined by one-way ANOVA multiple comparisons among means, with the Turkey's *post hoc* test. For survival analysis, the data were compared with a Kaplan–Meier curve and log-rank test. Results were considered statistically significant when *P*<0.05.
