# Supplementary material for: Endogenous AMPK acts as a detrimental factor in fulminant hepatitis via potentiating JNK-dependent hepatocyte apoptosis
Source: Cell Death Dis. 2017 Mar 2;8(3):e2637–. doi: 10.1038/cddis.2017.62 (PMC5386558; doi:10.1038/cddis.2017.62)
Supplement: Supplementary Information [file cddis201762x1.doc]

**Supplementary Information**

**Supplementary Figure 1. Pretreatment with the AMPK activator A-769662 alleviated LPS/D-Gal-induced liver injury.** Mice were exposed to LPS/D-Gal with or without various doses of AMPK activator A-769662 pretreatment, the liver and plasma samples were harvested at 6 h after LPS/D-Gal exposure. (a) The levels of ALT in plasma were determined, n=8. (* *P*<0.05, ** *P*<0.01) (b) Liver sections were stained with hematoxylin & eosin for morphological evaluation and the representative liver sections of each group are shown.

**Figure 2. Pretreatment with the AMPK activator A-769662 suppressed LPS/D-Gal-induced early inflammation.** Mice were exposed to LPS/D-Gal with or without the AMPK activator A-769662 pretreatment, the liver and plasma samples were harvested at 1.5 h after LPS/D-Gal exposure. (a) The phosphorylated AMPKα and the total level of AMPKα were determined by Western blot analysis, n=4; (b) The plasma levels of TNF-α were determined by ELISA, n=8. (^NS^ *P*>0.05, * *P*<0.05, ** *P*<0.01)
