# Supplementary figures and images for: Endogenous AMPK acts as a detrimental factor in fulminant hepatitis via potentiating JNK-dependent hepatocyte apoptosis
Source: Cell Death Dis. 2017 Mar 2;8(3):e2637–. doi: 10.1038/cddis.2017.62 (PMC5386558; doi:10.1038/cddis.2017.62)

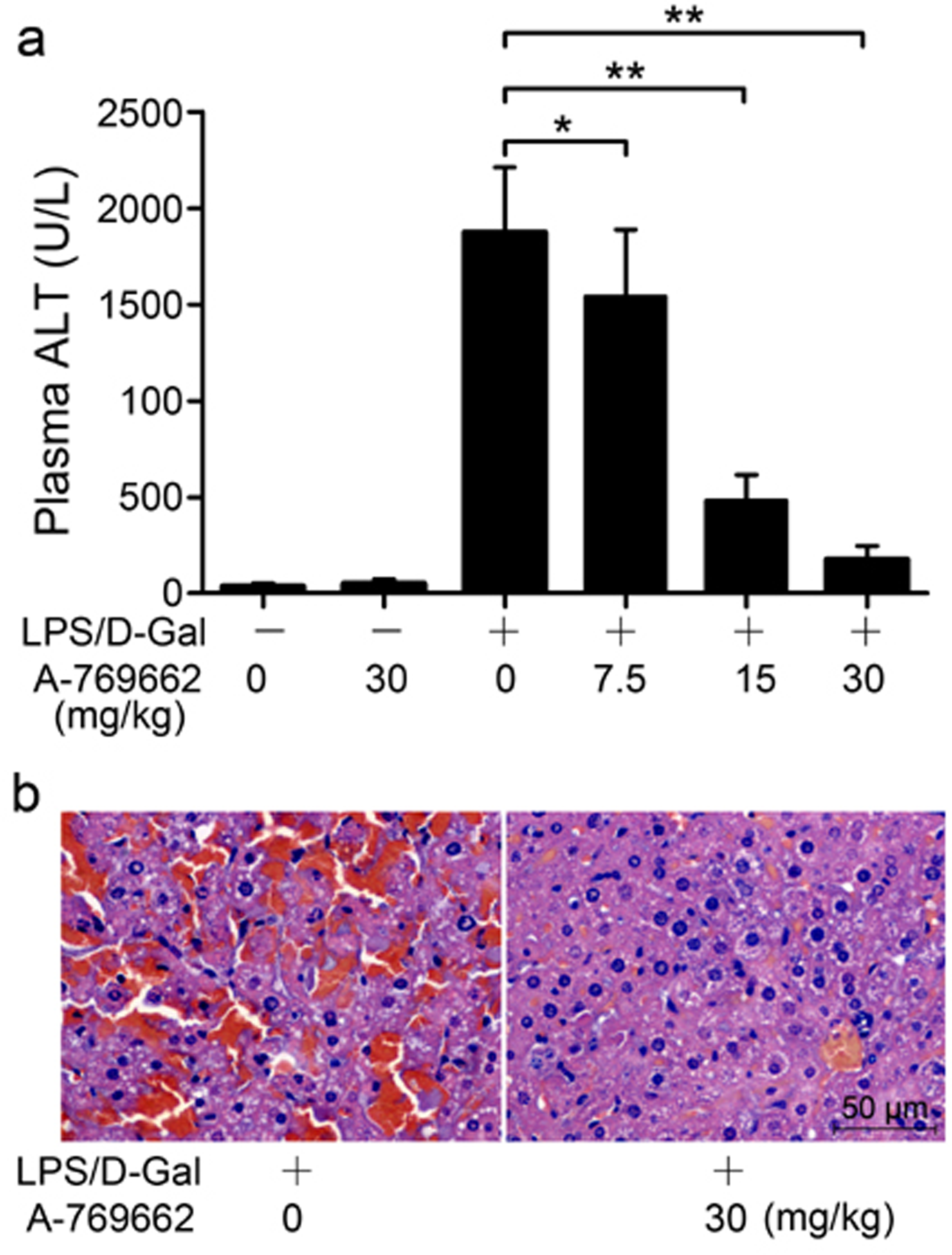

Supplement: Supplementary Figure 1 [file cddis201762x2.tif]

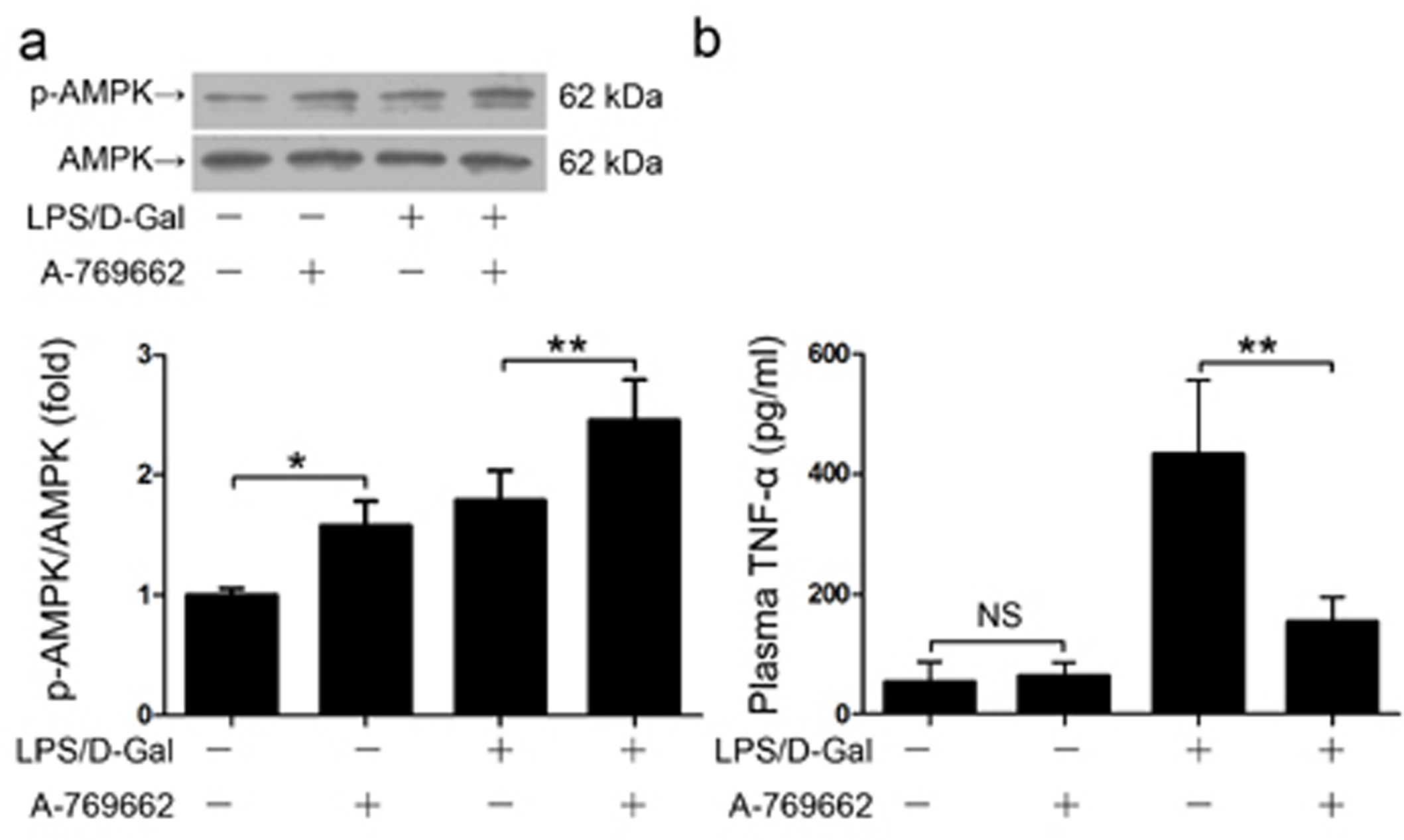

Supplement: Supplementary Figure 2 [file cddis201762x3.tif]
